# The orthologous Tbx transcription factors Omb and TBX2 induce epithelial cell migration and extrusion *in vivo* without involvement of matrix metalloproteinases

**DOI:** 10.18632/oncotarget.2426

**Published:** 2014-09-02

**Authors:** Jie Shen, Juan Lu, Liyuan Sui, Dan Wang, Meizhen Yin, Inka Hoffmann, Anne Legler, Gert O. Pflugfelder

**Affiliations:** ^1^ Department of Entomology China Agricultural University, Beijing, China; ^2^ Key Laboratory of Carbon Fiber and Functional Polymers, Beijing Laboratory of Biomedical Materials, Beijing University of Chemical Technology, Beijing, China; ^3^ Institute of Genetics, Johannes Gutenberg-University, Mainz, Germany

**Keywords:** TBX2, invasion, E-cadherin, extracellular matrix, Drosophila wing epithelium, optomotor-blind

## Abstract

The transcription factors TBX2 and TBX3 are overexpressed in various human cancers. Here, we investigated the effect of overexpressing the orthologous Tbx genes *Drosophila optomotor-blind* (*omb*) and human *TBX2* in the epithelium of the *Drosophila* wing imaginal disc and observed two types of cell motility. Omb/TBX2 overexpressing cells could move within the plane of the epithelium. Invasive cells migrated long-distance as single cells retaining or regaining normal cell shape and apico-basal polarity in spite of attenuated apical DE-cadherin concentration. Inappropriate levels of DE-cadherin were sufficient to drive cell migration in the wing disc epithelium. Omb/TBX2 overexpression and reduced DE-cadherin-dependent adhesion caused the formation of actin-rich lateral cell protrusions. Omb/TBX2 overexpressing cells could also delaminate basally, penetratingthe basal lamina, however, without degradation of extracellular matrix. Expression of Timp, an inhibitor of matrix metalloproteases, blocked neither intraepithelial motility nor basal extrusion. Our results reveal an MMP-independent mechanism of cell invasion and suggest a conserved role of Tbx2-related proteins in cell invasion and metastasis-related processes.

## INTRODUCTION

The closely related T-box transcription factors TBX2 and TBX3 are frequently overexpressed in melanoma [1-3] and various types of human cancers such as breast, bladder, liver, and pancreas carcinoma [4-7]. TBX2/3 impinge on different cellular mechanisms to promote tumorigenesis. TBX2/3 can stimulate proliferation [8-13] and can contribute to transformation by suppressing senescence and anoikis [2, 14-18]. TBX2/3 can promote epithelial-mesenchymal transition (EMT) and invasive cell behavior in melanoma and breast cancer cells [3, 7, 13, 19]. In colorectal cancer, TBX2 overexpression correlates with poor prognosis [20]. TBX2/3 may also contribute to breast cancer growth by promoting the proliferation of cancer stem-like cells [9, 21].

TBX2 and TBX3 are members of the Tbx2 subfamily of T-box transcription factors [22]. They are important developmental regulators controlling, among others, the development of heart, limbs, the visual system, and mammary tissue [5, 23-28]. TBX3 haploinsufficiency in human causes Ulnar-mammary syndrome [29, 30].

In *Drosophila*, the only Tbx2 subfamily gene is *optomotor-blind* (*omb*). Like its vertebrate orthologs, Omb controls many developmental processes [31]. Of these, wing development has been studied most closely. The adult wing develops from a simple larval epithelial tissue, the wing imaginal disc. This consists of two opposing epithelial cell layers: the peripodial epithelium (composed of squamous cells) and the disc proper or main epithelium (composed of columnar cells). The adult wing is largely derived from the main epithelium. Omb is expressed in most cells of the future wing blade [32] and is essential for correct cell morphogenesis [33], epithelial integrity [34], growth control [35], and pattern formation [36]. *Drosophila* is a recognized model for investigating etiology and treatments of mutation-based human disorders, including slowly progressing diseases such as neurodegeneration and tumorigenesis [37-46]. The structural simplicity of *Drosophila* imaginal disc epithelia and the ease with which these can be manipulated genetically allow detailed studies of the molecular and cellular processes leading to transformation (e.g. [47, 48]).

We here investigated the consequences for cellular behavior of overexpressing Omb and TBX2 in the wing disc epithelium. Our results show that *omb* overexpression can induce intraepithelial cell motility. Omb overexpressing cells could also delaminate basally from the epithelium, thereby penetrating the extracellular matrix (ECM). Overexpression of Omb and TBX2 caused an attenuation of apical DE-Cadherin.

## RESULTS

### omb overexpressing cells can translocate long distance in the wing disc epithelium

**Figure 1:**
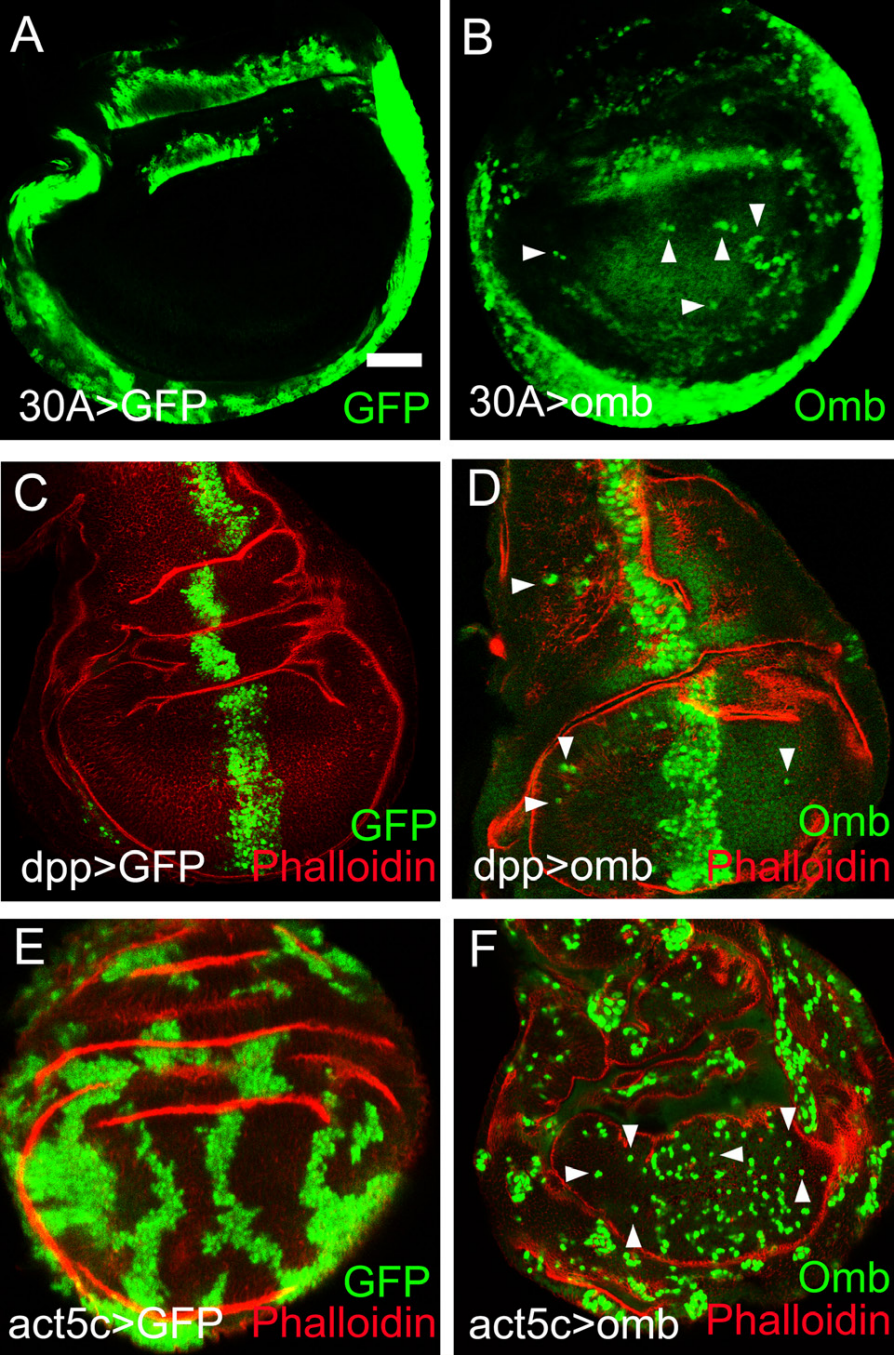
Long distance migration of *omb* overexpressing cells in the *Drosophila* wing imaginal disc In this and subsequent figures, wing imaginal discs were oriented anterior left and dorsal up. The developmental stages of the wing imaginal discs were middle to late third instar, unless indicated otherwise. x-y images were scanned at middle sections and were focussed on the wing pouch region, unless indicated otherwise. Scale bars are 50 μm. (A) Cells expressing GFP (green) in the 30A-Gal4 domain did not migrate out of their expression domain. In this and all subsequent panels, the symbol “>” denotes the connection between Gal4 driver and UAS regulated genes. Hence, 30A>GFP is short for 30A-Gal4, UAS-GFP. (B) Cells expressing Omb (green) in the 30A-Gal4 domain migrated into the central wing pouch. Cells derived from the 30-Gal4 domain were defined by high level Omb expression (intense green, arrowheads). The comparatively uniform and lower fluorescence intensity in the centre of the wing disc visualized the endogenous Omb expression. (C) Dpp>GFP cells (green) were confined to the *dpp* expression domain. (D) Cells overexpressing Omb (intense green) in the dpp-Gal4 domain migrated long distance away from the central expression stripe both into the anterior and posterior compartment (arrowheads). (E) Clonal cells expressing GFP (green) were clustered in groups. (F) Clones of cells overexpressing Omb (intense green) tended to disperse to the single cell level. In (C - F) wing discs were counterstained with Rhodamin phalloidin (red) which highlights folds in the epithelium.

Genetically normal (wild type) cells exhibit little motility in the wing disc epithelium. This is apparent from the expression pattern of enhancer traps which cell-autonomously render the activity of the “trapped” genes [49]. When the expression patterns of Gal4 lines are visualized by the fluorescent marker protein GFP, expression domains tend to have well defined spatial borders (Fig. 1A, C). This was not the case when *omb* expression was driven by the same Gal4 inserts (Fig. 1B, D). 30A-Gal4 is expressed in a ring around the wing pouch, in cells of the future hinge and pleura (Fig. 1A). In 30A>omb discs, Omb overexpressing cells could be found outside the 30A domain scattered within the wing pouch (Fig. 1B). In Fig. 1B (as well as in Fig. 1D, F) Omb overexpressing cells are identified by their higher expression level which can be recognized above the more uniform background of the endogenous Omb expression (cf. [33]). The observation of Omb overexpressing cells in the central region of the wing disc suggests that they migrated in from the periphery. In order to determine whether migration was directed, we overexpressed *omb* in the central wing region by the dpp-Gal4 driver. In this case, Omb overexpressing cells were found in both anterior and posterior regions far away from the *dpp* expression domain (Fig. 1C and D) indicating centrifugal motility with this Gal4-driver. Enhanced cell motility was also observed when *omb* overexpressing cells were generated randomly in clones by flippase-induced recombination [50]. In control clones, cells descending from one progenitor tended to remain clustered, even though the rugged clone outlines show that the clonal cells differed little in adhesive properties from their unmarked neighbours (Fig.1 E). When *omb* was overexpressed in clones, grouped clonal cells were rare. The majority of clonal cells were dispersed to the single cell level (Fig. 1F). Motility appeared enhanced in the clonal expression experiment (Fig. 1F) compared to the regional *omb* overexpression experiments (Fig. 1B and D). This is a consequence of the experimental design. In the cell clone experiment, omb overexpression is generated by flipping out the stop cassette ( the *yellow* gene) from an Act5c>yellow>Gal4 (AYGal4) construct. The “>” symbols here denote flippase recombination target (FRT) sequences. Recombinant cells and all their clonal daughter cells will, therefore, stably express UAS-omb under the control of Actin5C-Gal4 which is uniformly and constitutively active in the wing disc (cf. the position-independent level of GFP fluorescence in Fig. 1E). Thus, in a migrating cell, the level of *omb* overexpression will remain constant independent of its position in the imaginal disc. This is different in experiments with Gal4 drivers whose activity is regionally restricted. For instance, when cells move out of the activity domain of the dpp-Gal4-driver into the lateral wing disc, the driver becomes inactive and *omb* overexpression is turned off. The detectability of overexpressed Omb in migrating cells is limited by the perdurance of the Omb protein.

When omb was overexpressed in the wing disc periphery (under control of 30A-Gal4, Fig. 1B) cells migrated into the center of the disc. When omb was overexpressed medially (under control of dpp-Gal4, Fig. 1D) cells migrated into the periphery of the disc. Hence, there was no indication of a single long-range attractive force acting on the *omb* overexpressing cells. 4D imaging will help to elucidate the forces which drive migration.

### Migrating omb overexpressing cells retain or regain their normal cell shape

**Figure 2:**
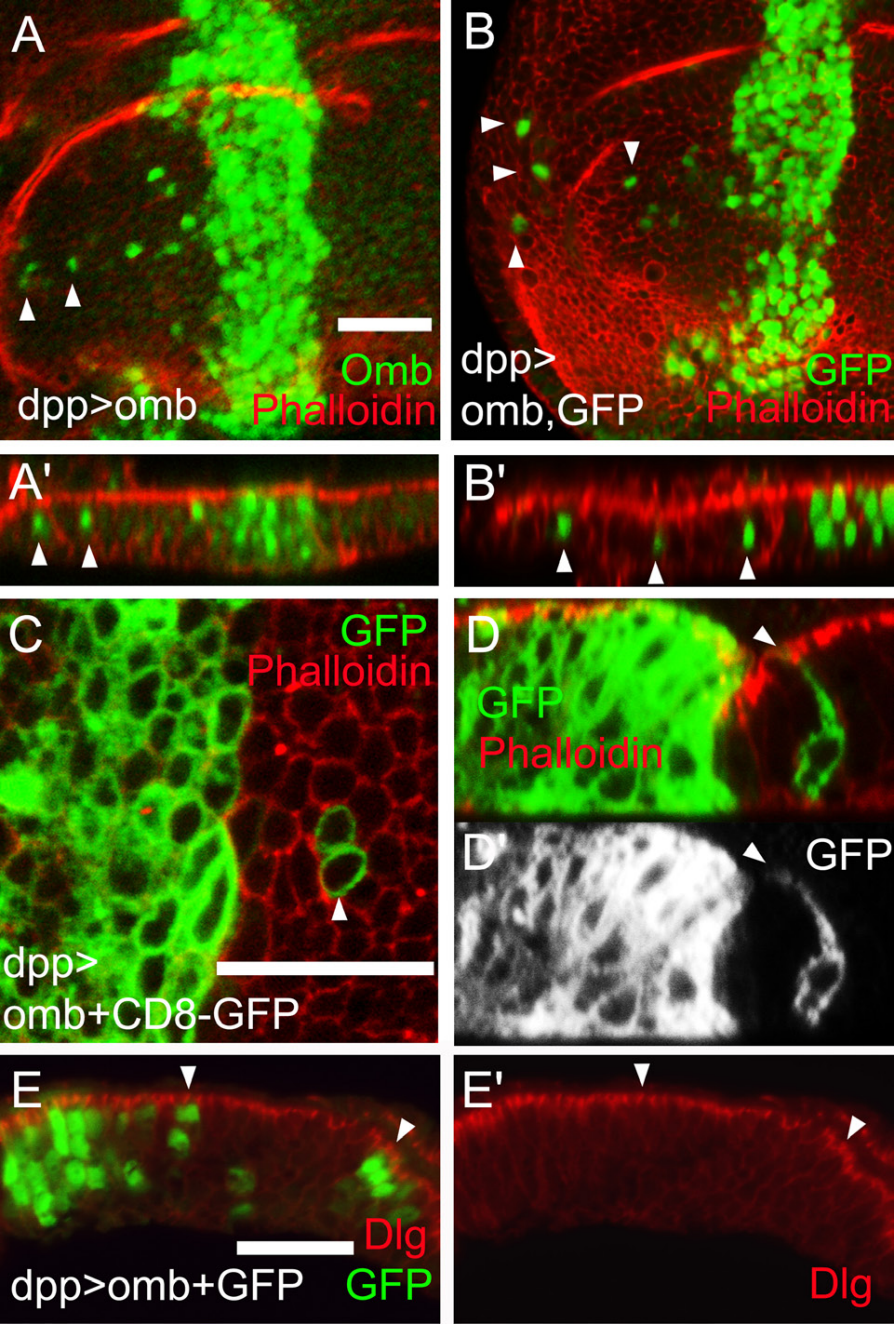
Apparently normal cell shape of migrated cells Cells overexpressing Omb (green) (A) or co-overexpressing *omb* with GFP (green) (B) in the dpp-Gal4 domain could migrate long distance to the periphery of the disc. In all x-z scans apical is up and anterior left. (A′ and B′) x-z scans of A and B. Nuclei of migrated cells had a wild-typic apico-basal position (arrowheads). (C and D) Cells co-overexpressing *omb* along with CD8-GFP (green) after crossing the A/P compartment boundary had wild-typic cell diameter (C) and apico-basal extension (D and D′). In A-D wing discs were counterstained with rhodamine phalloidin (red) highlighting cortical filamentous actin. (E) Cells that overexpressed *omb* (green) did not affect the subcellular distribution of Dlg (red), neither in the *dpp* expression domain (at the left end of panel) nor in migrated cells (arrowheads).

Omb overexpressing cells could migrate over a distance of more than a dozen cell diameters up to the edge of the wing disc pouch (Fig. 2A and B). When the discs were inspected in x-z scans, it was obvious that the nuclei of migrated cells occupied normal positions in the pseudostratified epithelium, outlined by phalloidin staining, and were not expelled toward the basement membrane (Figure 2A′ and B′). In Fig. 2A′ this is apparent when comparing the position of Omb overexpressing nuclei (intense green) to that of nuclei expressing Omb at the endogenous level in the adjoining cells (faint green). To reveal the shape of the migrated cells, we co-expressed the membrane marker CD8-GFP with *omb*. Migrated cells were normal in cell diameter and shape (Fig. 2C). In particular, the migrated cells spanned the apico-basal width of the epithelium (Fig. 2D). To further test the apico-basal polarity in migrated cells, we stained against Discs large 1 (Dlg1). Dlg1 in complex with Scrib and Lgl is essential for septate junction formation and apico-basal polarity [51]. The genes encoding these proteins are tumor suppressor genes [52] [42]. The level and distribution of Dlg1 was unaffected by Omb overexpression both in the dpp-Gal4 expression domain and in migrated cells (Fig. 2E). We cannot rule out that, in the actual process of movement, the migrating cells adopt a different conformation. We can conclude, however, that even after having migrated long distance, the *omb* overexpressing cells either retained or resumed normal epithelial cell shape.

### Omb overexpression can cause basal delamination

**Figure 3:**
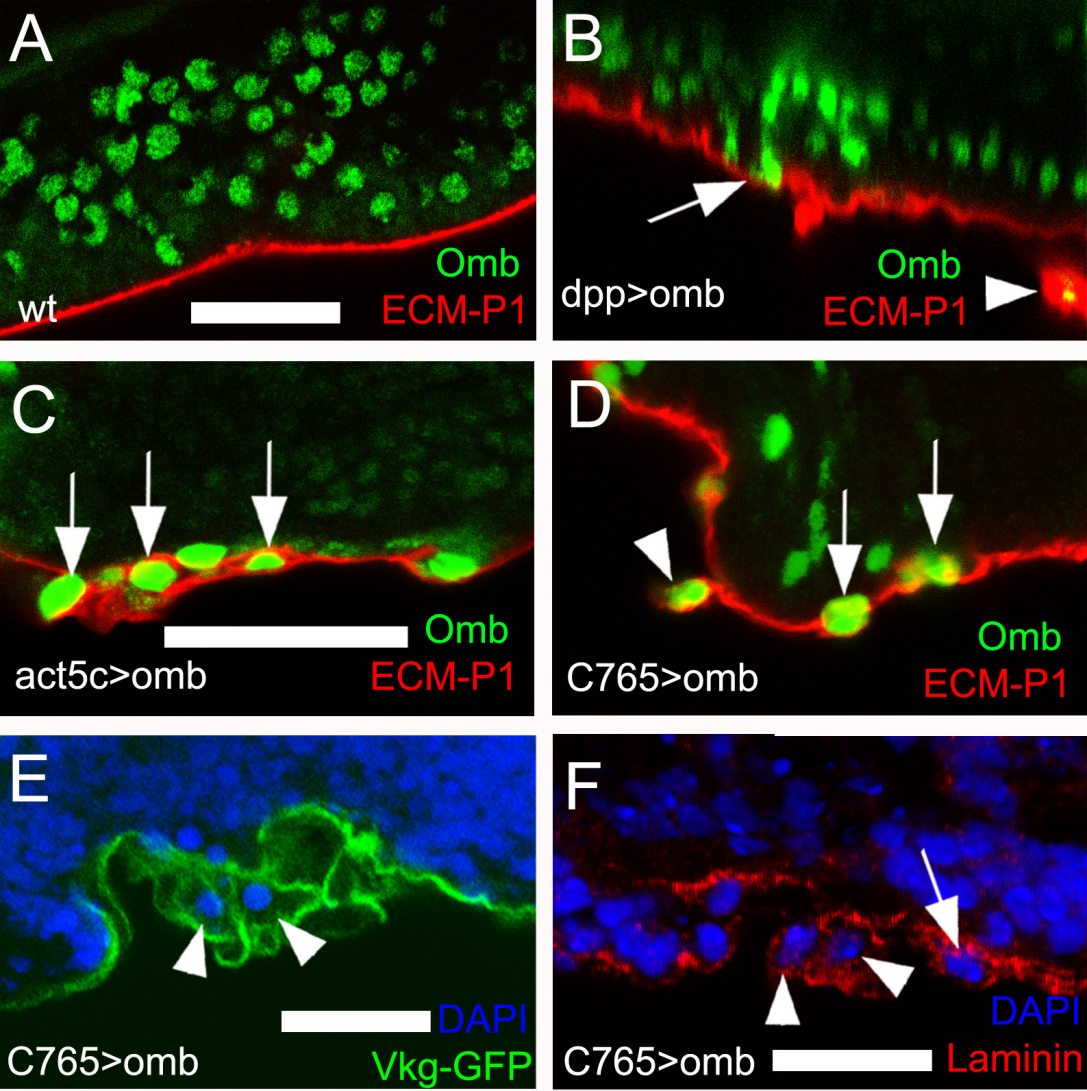
*Omb* overexpressing cells penetrate the ECM ECM was stained by the fluorescent core-shell macromolecule ECM-P1 (red), Vkg-GFP (green), and anti-Laminin (red), respectively. All panels are x-z scans. (A) and (B), and (C) and (D) are at the same magnification, respectively. (A) ECM-P1 dye specifically stained the basement membrane of wild type wing disc. (B) Individual dpp>omb cells (green) apparently could penetrate the ECM (arrow). In such preparations, ECM-clad cells outside the basement membrane were observed (arrowhead). (C) Cells overexpressing Omb (green) derived from Act5C>omb clones could penetrate the ECM (arrows). (D-F) *omb* was overexpressed by the imaginal disc-specific driver C765-Gal4. Arrowheads and arrows indicate *omb* overexpressing cells penetrating the ECM (arrowheads) or located peripheral to the basement membrane (arrows).

In addition to the intraepithelial motility described above, we observed cells that basally delaminated from the epithelium, penetrating the basal lamina, thus entering the body cavity. We used three types of molecular markers to visualize the ECM of the basal lamina. ECM-P1 is a highly positively charged fluorescent dye that selectively binds to the negatively charged ECM [53] (Fig. 3A-D). The type IV collagen Viking was visualized as a GFP-protein trap [54] (Fig. 3E), laminin by antibody binding [55] (Fig. 3F). At the late third instar stage, individual Omb overexpressing cells originating from the dpp-Gal4 expression domain could become extruded toward the basal side of the epithelium, apparently penetrating the ECM (Fig. 3B, arrow). In such preparations, ECM-clad cells outside the basement membrane could be observed that had largely lost contact with the epithelium (Fig. 3B, arrowhead). A similar phenotype was observed when *omb* was overexpressed clonally. Here, too, cells embedded in and partly emerging from the ECM could be observed (Fig. 3C, arrows). To rule out the possibility that cells outside of the disc epithelium were of non-disc origin, we overexpressed *omb* under control of the disc-specific driver C765-Gal4 [56]. Also with this driver, partially (arrows) or fully extruded (arrowhead) Omb overexpressing cells were seen (Fig. 3D - F).

**Figure 4:**
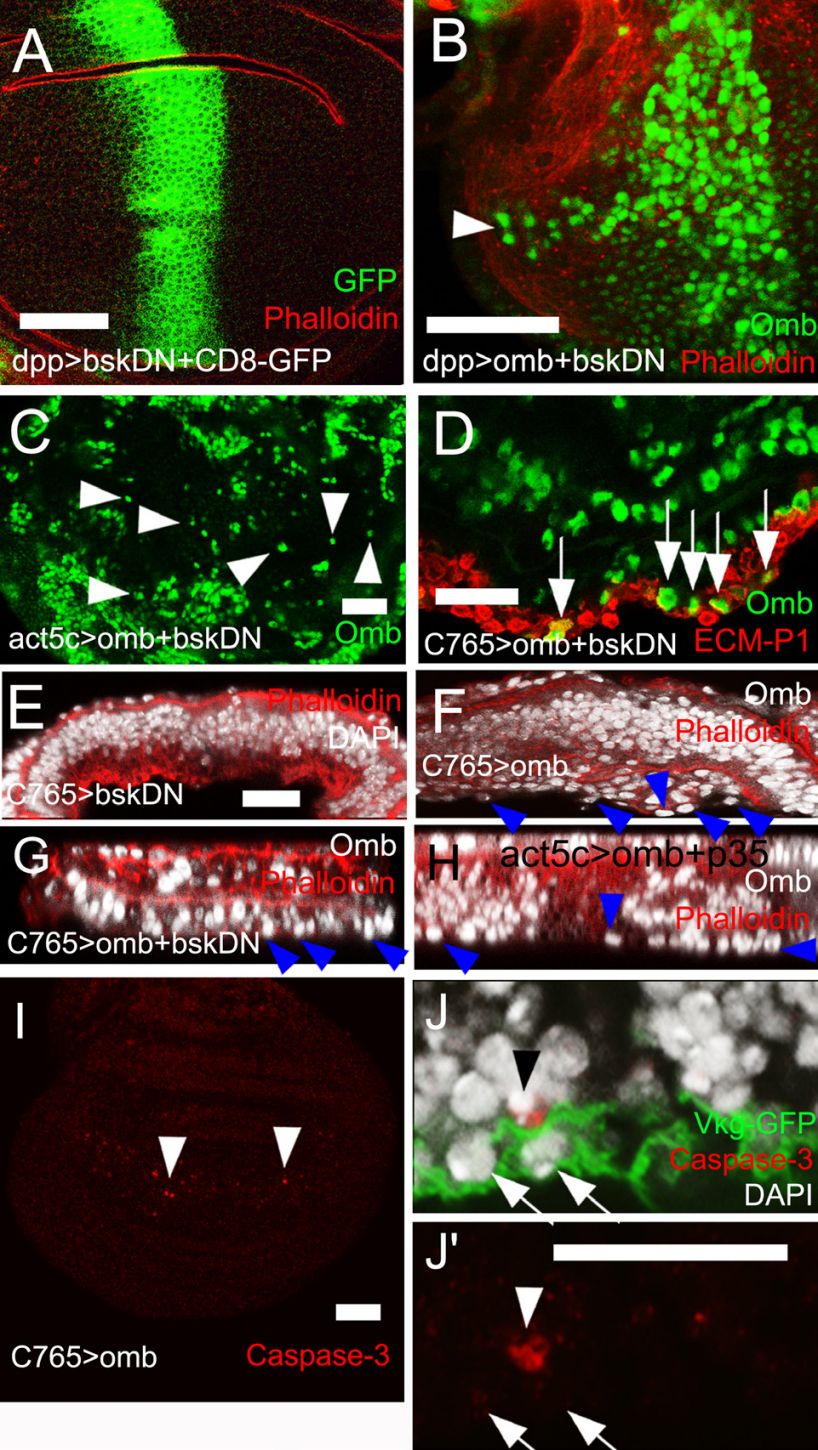
JNK signalling and apoptosis are not required for *omb*-induced cell motility (A) Expression of *bsk[DN]* did not elicit cell motility. *Bsk[DN]*-expressing cells are marked by co-expression of GFP (green). (B) Cells co-overexpressing *omb* and *bsk[DN]* in the *dpp-Gal4* domain were still motile (arrowhead). (C) Clone cells co-overexpressing *omb* and *bsk[DN]* were dispersed to single cells (arrowheads). (D) - (H) and (J, J′) are x-z scans. (D) Cells co-overexpressing *omb* and *bsk[DN]* still invaded the basal ECM (arrows). (E) Ubiquitous expression of *bsk[DN]* did not cause basal delamination. (F) Disc-wide overexpression of *omb* induced extrusion of cells (arrowheads) which was not prevented by co-expression of *bsk[DN]* (G) or p35 (H). (I) Ubiquitous *omb* overexpression caused little caspase-3 activation (arrowheads). (J) Ubiquitous *omb* overexpression (C765>omb) induced basal invasion (arrows) not associated with anti-Caspase staining. A rare P-cas-3 positive cell is also contained in the picture (arrowhead). In (A), (B), and (E) - (H) discs were counterstained with rhodamine phalloidin (red).

Sharp spatial discontinuities in Omb level can cause JNK-dependent apoptosis [33, 57]. To test whether JNK activation is required for Omb-induced migration and extrusion, a dominant-negative form of JNK (*bsk[DN]*, [58]) was co-expressed with *omb*. This neither blocked migration (Fig. 4B and C) nor penetration of the ECM (Fig. 4D,G). Such discs, when inspected in x-z cross-section, showed an accumulation of cells underneath the basal membrane, probably due to reduced cell death in this genotype [59] (Fig. 4F). Expression of *bsk[DN]* alone did not elicit intraepithelial motility or delamination (Fig. 4A, E). In order to directly monitor JNK pathway activation we looked at the expression of the JNK target gene *puckered* (*puc*) [60] under conditions in which Omb induces cell motility. Puc-lacZ was not induced (SFig. 2A, B). Overexpression of the small GTPase Rho1 in the wing disc can cause invasive cell behavior [61] and was shown to act upstream of JNK [62, 63]. However, Omb overexpression, did not cause increased Rho1 expression (SFig. 2D). Therefore, *omb* overexpressing cells neither activate nor require JNK pathway activity for invasive behavior.

Fit neighbors can extrude apoptotic cells out of the epithelium [64]. To suppress potential cell death that could be induced by sharp discontinuities in Omb level, the anti-apoptotic gene *p35* [65] was co-expressed with *omb* in clones. Delamination still occurred (Fig. 4H). Recently, activation of the initiator caspase was found sufficient to induce cell invasion when execution of apoptosis was blocked by p35 expression [66]. To further rule out the possibility of apoptotic or caspase-induced extrusion, *omb* was ubiquitously overexpressed in C765>omb wing discs to avoid sharp spatial discontinuities in Omb level. Cross-sections showed only little cell death in this genotype (Fig. 4I, arrowhead) but penetration of the ECM could still occur (Fig. 4J). In order to completely block the apoptotic pathway, we co-expressed the general caspase inhibitor Diap1 [67] with omb. This did not prevent basal delamination (SFig. 2C) indicating that Omb-induced cell death and extrusion are separable events.

### Omb overexpression causes down-regulation of apical DE-cadherin

**Figure 5:**
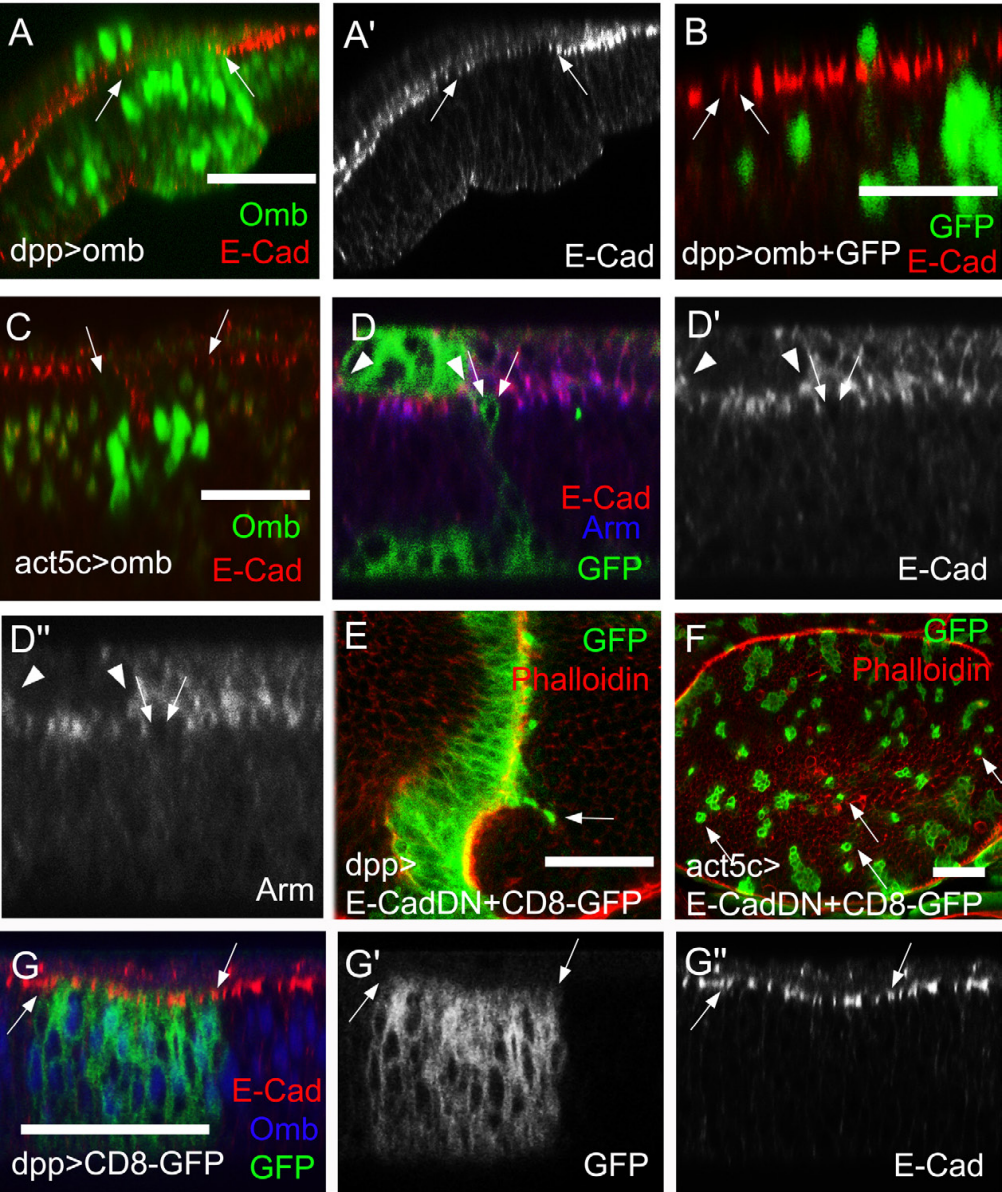
DE-Cad and Arm levels are reduced in *omb* overexpressing cells (A-D′) and (G-G″) are x-z scans. (A) When Omb (green) was overexpressed in the dpp-Gal4 domain, the DE-cad level (red) was attenuated (between arrows). (B) Migrated *omb* overexpressing cells (green) exhibited a reduced apical DE-cad level (arrows). The single green nucleus lying apical to the band of E-cad staining is in the peripodial membrane where dpp-Gal4 is also active. (C) When *omb* was expressed in clones, the DE-cadherin level was attenuated in the range of the clone. (D-D″) *omb* was co-expressed with CD8-GFP in clones (green). Single *omb* overexpressing and non-extruded cell had reduced apical DE-cad (red) and Arm (blue) levels. This also held for the clone in the periopodial epithelium outlined by arrowheads. (E) Cells expressing dominant negative DE-cad in the dpp-Gal4 domain could migrate across the A/P compartment boundary (arrow). (F) Clones of cells expressing dominant negative DE-cad (green) tended to disperse to the single cell level (arrows). Discs in (E) and (F) were counterstained with rhodamine phalloidin (red). (G) In a control wing imaginal disc expressing only the plasma membrane marker (CD8-GFP, green), E-cad expression (red) was uniform in- and outside the dpp-Gal4 expression domain, marked by arrows. The disc was counterstained against Omb (blue).

Invasive motility generally is associated with down-regulation of the apical cell adhesion junction molecules E-cadherin (DE-cad or Shotgun, Shg) and β-catenin (Armadillo, Arm) which can result in reduced epithelial stability [68]. *Omb* overexpression in the dpp-Gal4 domain caused local attenuation of apical DE-cad (Fig. 5A,′. Compare Fig. 5G for DE-cad expression in a wild type wing disc). This also held for individual cells that had migrated out of the dpp-Gal4 domain. Migrated dpp>omb cells showed a reduction of the apical DE-cad level (Fig. 5B, arrows indicate the apical position of a migrating cell). Attenuation of DE-cad was also seen in *omb* overexpressing clones (Fig. 5C). To better visualize the cellular outline of the *omb* overexpressing cells, we co-expressed the membrane marker CD8-GFP. This showed that in the apical range of the single cell, which still maintained apico-basal polarity, both DE-cad and Arm were down-regulated (Fig. 5D). These results suggest that *omb* overexpression can weaken cell-cell interactions which generally remain strong enough, however, to allow cells to maintain apparently normal apico-basal cell shape.

To test the significance of the reduced DE-cad level for the migratory phenotype of *omb* overexpressing cells, we co-expressed *DE-cad* along with *omb*. Long-distance cellular migration could still be observed (Fig. S1 A, arrowheads). However, this observation is difficult to interpret because overexpression of *DE-cad* alone induced both anterior and posterior migration (Fig. S1B, arrowheads). On the other hand, expression in the dpp-Gal4 domain of a dominant negative form of DE-cad (DE-cad[DN] [69]) was sufficient to induce cell migration across the A/P boundary (Fig. 5E) or to cause cellular dispersal when it was clonally overexpressed (Fig. 5F). Therefore, both down- and up-regulation of DE-cad could cause cell migration in the wing disc epithelium. Thus, in *omb* overexpressing cells, down-regulation of DE-cad is likely to contribute to cellular motility.

### Actin-rich cellular protrusions in migratory omb overexpressing cells

**Figure 6:**
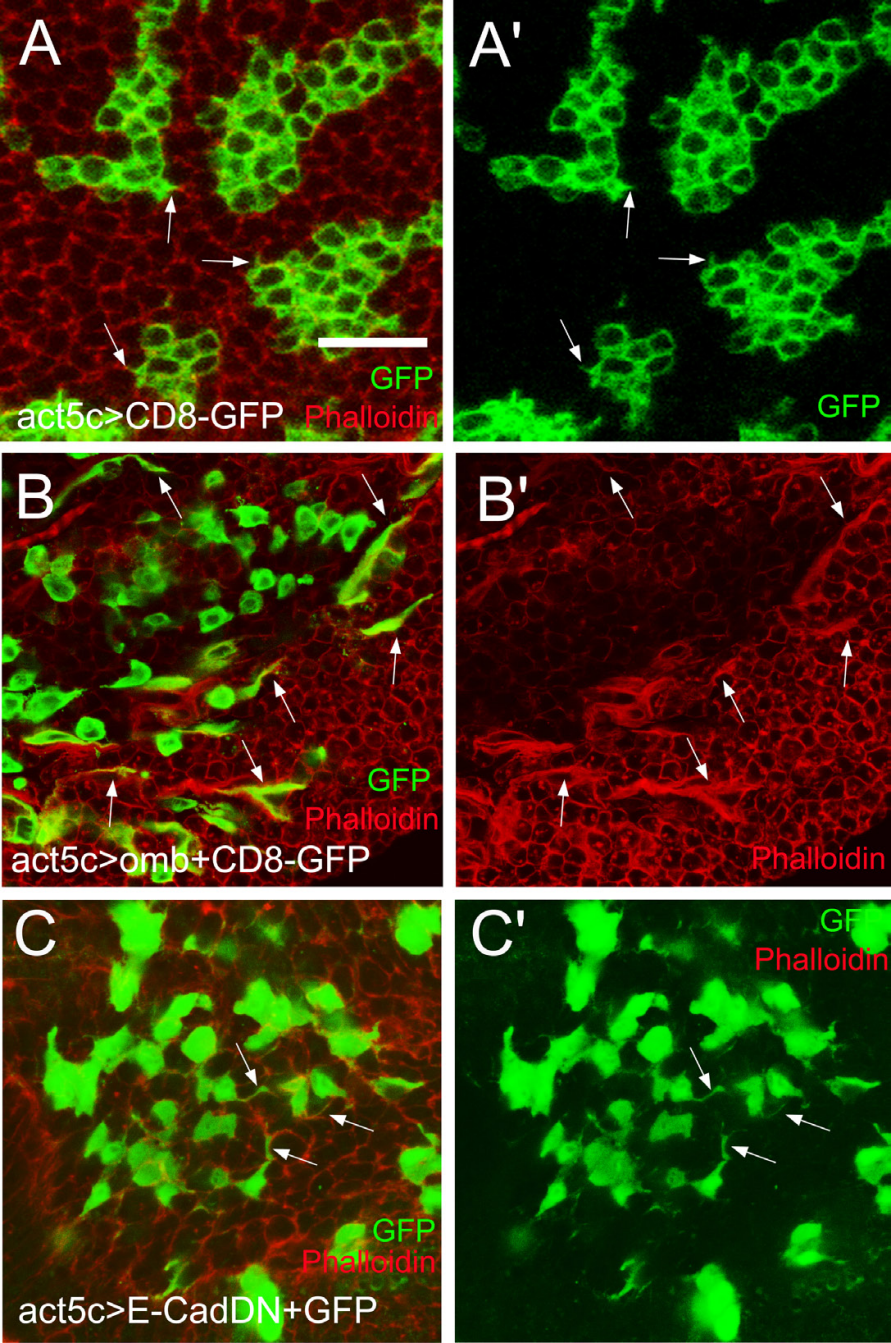
Actin-rich cellular protrusions in migratory *omb* overexpressing cells (A and A′) Control CD8-GFP clones with short lateral protrusions (arrows). (B and B′) Omb-overexpressing cells, visualized by co-expression with the membrane marker CD8-GFP, were dispersed and frequently exhibited long lateral protrusions enriched in F-actin (arrows). (C and C′) Expressing a dominant negative form of DE-Cad induced long lateral protrusions (arrows). Filamentous actin was visualized by counterstaining with rhodamine phalloidin (red).

Consistent with a previous report [70], short protrusions, less than one cell diameter in length, were detectable in control clones labelled by CD8-GFP expression (Fig. 6A, arrows), although at low penetrance under normal fixation conditions. Omb overexpression caused many cells in Act5C>omb discs to extend actin-enriched protrusions in the plane of the epithelium that were longer than one cell diameter. Protrusion-bearing cells tended to be elongated along the A-P axis of the wing pouch, whereas cells lacking protrusions were roundish (Fig. 6B). Widmann and Dahmann generated null mutant DE-cad clones in the wing pouch and found that mutant cells were retracted or extruded toward the basal membrane and could exhibit actin-rich protrusions [71]. Consistent with their results, overexpressing DE-cad[DN] caused cellular dispersal and induced long lateral protrusions (Fig. 6C). Since such cellular extensions are characteristic of migratory cells, it appears that attenuation of DE-cad function is crucial for the cell biological effects of *omb* overexpression.

### Expression of human Tbx2 in the Drosophila wing disc induces long distance migration and down-regulation of cell adhesion

**Figure 7:**
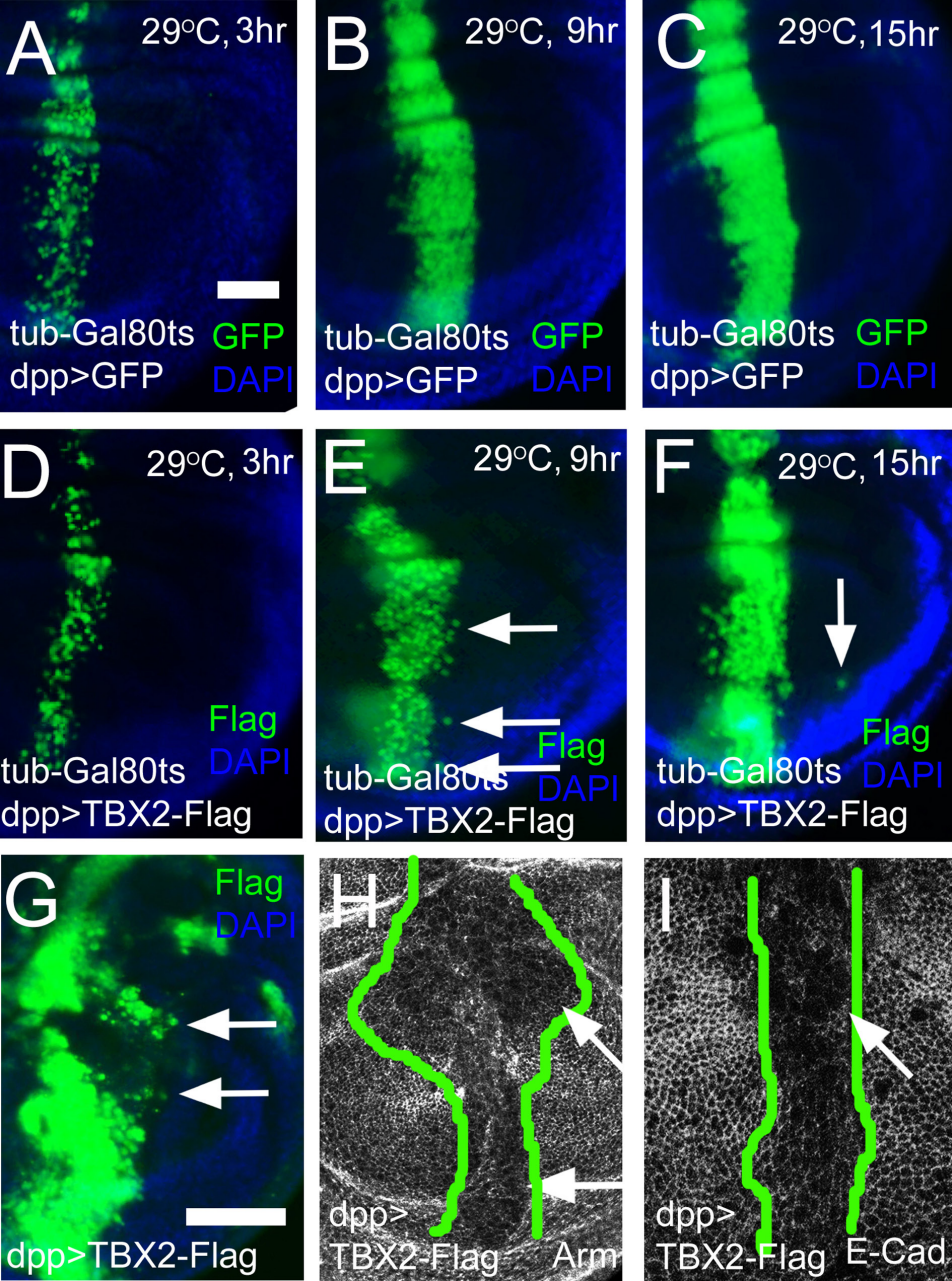
Expression of human TBX2 induces long distance migration and down-regulates adhesion junction molecules (A-C) Time course of GFP expression (green) in the dpp-Gal4 domain. The hours after inactivation of Gal80^ts^ by temperature upshift are indicated. (D-F) Time course of human TBX2-Flag (green) expression in the dpp-Gal4 domain. Nine hours after induction, TBX2 expressing cells had started to migrate across the A/P boundary (E, arrows). After 15 hours of induction, TBX2 cells were found at a larger distance from the dpp-Gal4 domain (F, arrow). (G) Constitutive expression of TBX2 in the dpp-Gal4 domain induced cells migration (arrows). In (A) - (G) discs were counterstained with DAPI (blue). (H and I) Apical sections showed the reduction of Arm (H) and E-Cad (I) in TBX2 expressing cells. The range of TBX2 expression is outlined by green lines based on the anti-Flag staining.

**Figure 8:**
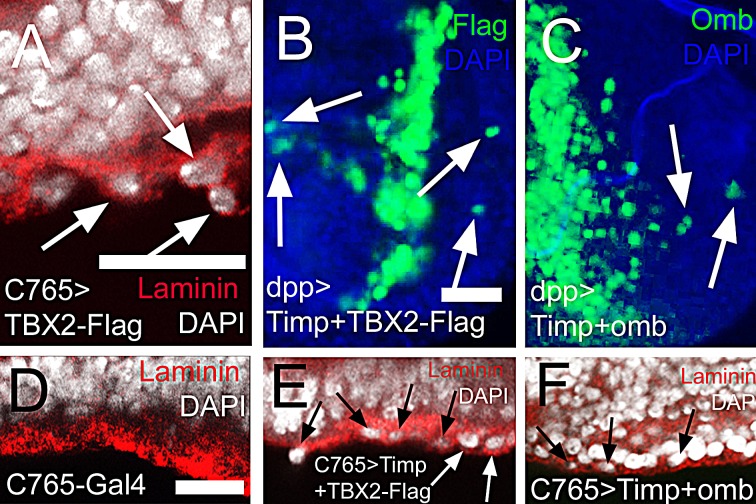
MMP activity is not required for intraepithelial motility and basal delamination induced by overexpression of TBX2 and Omb In (A) and (D) - (F) nuclei are counterstained with DAPI (white); In (B) - (C) DAPI is blue. (A) Expressing TBX2 induced invasion and penetration of the basement membrane (arrows) leading to externalized cells coated with laminin (red). (B, C) Co-expressing Timp with TBX2 (B) or Omb (C) did not prevent long distance migration (arrows). (D) Wild type control in x-z scan. Wild-typic cell nuclei were not observed within the basement membrane of the epithelium. (E, F) Co-expressing Timp with TBX2 (E) or Omb (F) did not prevent basal delamination.

TBX2 and TBX3 are the human orthologs of Omb [22]. As in the case of Omb, dpp-driven TBX2 expression caused long distance cell migration across the A/P boundary (Fig. 7G). In order to determine the rate at which TBX2 overexpressing cells moved through the epithelium, we employed an inducible genetic system. The Gal80^ts^ Gal4-UAS system allows temperature control of UAS target gene expression [72]. Transgene expression was induced by upshift from 18°C to 29°C. After TBX2 was induced for 9 hours, the first cells were observed to migrate out of the *dpp* expression domain (Fig. 7E, arrows). Long distance migration was observed at 15 hours (Fig. 7F). In the control experiment, no migration out of the dpp-Gal4 domain was apparent (Fig. 7A-C). As in the case of Omb, expression of TBX2 caused apical depletion of DE-cad and Arm (Fig. 7H and I), suggesting an essential and conserved association between cell migration and reduced cell-cell adhesion. Like Omb, TBX2 could induce cells to penetrate the ECM of the basement membrane. With TBX2, too, the extraepithelial cells were clothed in a coat of ECM (Fig. 8A).

### Intraepithelial motility does not require matrix metalloproteinase activity

In many experiments that used the imaginal disc model to investigate determinants of cellular motility an involvement of matrix metalloproteinases (MMPs) has been demonstrated. *Drosophila* encodes two MMP proteins, both of which are inhibited by tissue inhibitor of metalloproteinases (Timp) [73, 74]. Timp effectively blocks distant metastasis in several experimental systems [75, 76]. In Omb overexpression experiments, neither *MMP1* nor *MMP2* was induced in the overexpression domain (SFig. 3). When TIMP was co-expressed with Omb or TBX2 in the wing disc, either in a spatially restricted pattern (dpp-Gal4) or ubiquitously (C765-Gal4), neither long distance cell migration (Fig. 8B, C) nor basal delamination from the wing disc epithelium (Fig. 8E, F) was blocked by Timp co-expression. The requirement of Timp for cell migration in *Drosophila*, thus, appears to depend on the genotype which drives migration and on the experimental assay and will be discussed below.

## DISCUSSION

### Long distance intraepithelial translocation of Omb/TBX2 overexpressing cells

As is apparent from the localized expression of marker genes, there is little intrinsic cell motility in the normal wing imaginal disc (Fig. 1A, C, E) [77]. However, overexpression of *omb* caused cells to migrate away from their original site. In this process, they could move to new positions, up to the periphery of the imaginal disc (>20 cell diameters). The driving force for this migration is unclear. Cells could move both medially and laterally indicating that cells do not move under the influence of a single long-range attractive force (Fig. 1B, D). When *omb* was clonally overexpressed under control of the strong *Act5C* promoter, cells were dispersed down to the single cell level (Fig. 1F).

In the absence of live imaging data, the detailed mode of migration remains speculative, but several facts could be established. Cells that had migrated to a distal position (relative to their original site) apparently had normal cell shape, both within the plane of the epithelium (Fig. 2C) and with regard to apico-basal polarity (Fig. 2D, E). Also the position of their cell nuclei did not differ from neighbouring non-migratory cells (Fig. 2A′, B′). When *omb* overexpression was generated clonally, a large part of the cells in the wing disc became motile. Of these, a considerable fraction was elongated in the plane of the epithelium and exhibited a single actin-rich protrusion (Fig. 6B). This migration was independent of MMP activity as it occurred even when Timp was co-expressed (Fig. 8C). A similar migratory behaviour was induced by the expression of human TBX2 (Fig. 7D-F; Fig. 8B).

The mechanistic interpretation that most easily agrees with observations in human tumor biology is that Omb/TBX2 overexpression causes a type of epithelial-mesenchymal transition (EMT). This is generally considered a prerequisite for the mobilization of individual cancer cells from the original epithelium. The roundish shape with single protrusion that *omb* overexpressing cells can adopt suggests amoeboid rather than fibroblast-like motility [78]. Furthermore, amoeboid motility of cancer cells, like the intraepithelial translocation of *omb* overexpressing cells, is independent of MMP activity [79, 80]. The leading cellular protrusion is likely to act as a pseudopodium, involved in force generation and chemical sensing [81] rather than as a invadopodium which characteristically is endowed with MMP activity and serves to proteolytically breach the basement membrane [82]. EMT is known to be reversible. In the standard scenario of metastasis, a disseminated cancer cell reintegrates into an ectopic target epithelium by a mesenchymal-epithelial reverting transition [83] whereby it may eventually effect a successful colonization [84]. In the framework of this view, the cells shown in Fig. 2C migrated to their ectopic position in an amoeboid fashion (cf. Fig. 6B) and then reverted to their original epithelial structure. Omb overexpressing cells that have migrated out of a spatially fixed Gal4 expression domain (as in 30A>omb and dpp>omb) will lose their high Omb level at a rate governed by the perdurance of Gal4 and Omb. It is conceivable that a reversion to normal Omb level also reverts the cellular phenotype.

The motogenic effect of Omb overexpression is unrelated to its effect on proliferation. Omb expression in the wing imaginal disc dampens proliferation medially while it enhances proliferation laterally [35]. However, Omb overexpression mobilized cells to a similar extent irrespective of their original position (compare Fig. 1B and 1D). Also in Ras^V12^-dependent *Drosophila* cancer models overproliferation was neither necessary nor sufficient for systemic metastasis [52, 85].

### Basal delamination (transmigration) of Omb/TBX2 overexpressing cells

Omb and TBX2 overexpression caused a second type of cellular motility: Basal cell retraction which could be associated with penetration of the basement membrane and invasion of the body cavity. We have reported previously that the creation of discontinuities in the normally smoothly graded Omb expression profile in the wing disc can cause cells to retract basally. This effect is not secondary to apoptosis because retraction still occurs when cell death is repressed by expression of p35 or a dominant-negative form of JNK [33]. However, penetration into and through the BM did not require spatially discontinuous Omb/TBX2 expression because it could also be elicited by uniform overexpression (Fig. 3D, 7A).

As will be discussed below, Omb-induced cellular motility apparently did not require the activity of matrix metalloproteinases. Omb/TBX2 overexpressing cells emerged from the basement membrane with a coat of ECM (Figs. 3, 8). This, too, suggests that Omb/TBX2 overexpressing cells have little exo-proteolytic activity directed against the ECM. The ECM coat may allow Omb/TBX2 overexpressing cells to escape from anoikis [86]. In *Drosophila*, the major ECM constituent, collagen IV, is produced by the fat body, an organ distinct from the imaginal discs [87]. It is not clear whether the ECM of Omb/TBX2 cells derive from this source or whether it is torn out of the penetrated basement membrane. In many cancers, an increased synthesis of ECM molecules is associated with enhanced metastatic potential [87, 88]. The ECM coat also allows to distinguish extruded Omb/TBX2 cells from externally adhering hemocytes which are recruited to lesions in the ECM in a JNK-dependent manner. Such hemocytes lack an ECM coat [89].

### Effectors of Omb/TBX2-induced cell motility

Reduction in E-cadherin concentration is a hallmark of EMT and cell invasion [90]. A decrease in E-cad not only attenuates the stability of apical adherens junctions but can also have transcriptional and other consequences [91-93]. Omb/TBX2 overexpression caused reduction of apical E-cad (Fig. 4, 7I). Similarly, β-catenin (Armadillo) which is attached intracellularly to E-cad, was reduced (Fig. 7H).

In melanoma, TBX2 (and its paralog TBX3) directly repress *E-cad* by binding to its promoter, causing enhanced invasiveness [3, 19]. Numerous other transcription factors were shown to induce EMT by transcriptional repression of *E-cad* [94, 95]. This indicates that E-cad reduction and EMT can be achieved through several independent pathways. Bioinformatic analysis of the *Drosophila E-cad* gene did not reveal high affinity binding sites for Tbx transcription factors [96] suggesting that its regulation by Omb may be indirect. Redmond et al. (2010) showed that TBX2 can repress the breast tumor suppressor gene NDRG1 by acting as a co-factor for the transcription factor EGR1 [8]. Similarly, we have shown that TBX2 and TBX3 can repress gene expression from the human papilloma virus 16 long control region in the absence of sequences related to the T-box consensus binding element [97]. Omb, therefore, may repress *E-cad* expression either as a co-factor or by de-repressing another transcriptional repressor. Apart from transcriptional regulation, several posttranscriptional mechanisms have been identified by which E-cad protein level and localization can be controlled [98-101].

Whether reduction of E-cad suffices to elicit invasive cell behaviour appears to depend on the experimental system. In Act5C>Ras^V12^; *scrib* clones, co-expression of full-length E-cad suffices to suppress systemic metastasis in *Drosophila*; co-expression of a truncated protein lacking the extracellular domain does not, indicating the relevance of adhesion and/or cell communication [85]. In other systems, reduction of E-cad is not sufficient to induce EMT or to promote metastasis indicating the involvement of other factors [102-104]. Co-expression of E-cad did not prevent intraepithelial motility of Omb overexpressing cells. However, overexpression of E-cad alone elicited cellular migration (Fig. S1). In fact, under certain genetic conditions cellular motility may require elevated levels of E-cad [105]. Whether downregulation of E-cad is required for Omb/TBX2 induced motility, therefore, cannot be determined. Such a requirement is suggested, however, by the observation that interference with E-cad function by the expression of a dominant negative version of E-cad sufficed to disperse cells. These cells also showed single actin-rich extensions similar to the ones observed in Omb overexpressing cells (Fig. 6C). Also in mammalian models of carcinogenesis, expression of E-cad[DN] was shown to cause invasive behaviour [68].

### Omb/TBX2-induced cell motility is independent of JNK and MMP activity

Jun N-terminal kinase (JNK) signaling has an ambivalent role in carcinogenesis, acting pro- or anti-oncogenically depending on context [106]. In *Drosophila*, the function of the single JNK gene has been studied intensively both in normal and oncogenic development. In normal development, JNK signaling regulates cellular motility to effect epithelial morphogenesis as in embryonic dorsal and pupal thoracic closure [107, 108] and in the invasion of the larval epidermis by peripodial epithelium and stalk during wing disc eversion [109]. Activation of JNK signaling is crucial for invasive growth in fly tumor models which are based on a combination of loss of apico-basal polarity and expression of an activated oncogene [62, 66, 75, 110-114].

However, in a different *Drosophila* metastatic tumor model, based on Notch activation and epistatic dysregulation [115], JNK signaling mediates the tumor repressive activity of the differentiation factor Atonal. Inhibition of JNK signaling can be sufficient to promote metastatic growth when the Notch pathway is continuously activated [116]. In mouse and human cancer cell lines, the mammalian Ato ortholog ATOH1 appears to act by a similar mechanism [117]. Similarly, in loss-of-function clones of the *frazzled*, the fly ortholog of *Deleted in Colon Cancer* (*DCC*) which have metastatic potential when not eliminated by apoptosis, downregulation of JNK promotes the invasive phenotype [118] (cf. also [119])

In the case of Omb overexpression, none of the two migratory processes we observed was dependent on JNK signaling. Both intraepithelial motility and basal delamination occurred in the presence of a dominant negative form of JNK (Fig. 4B-D,). Co-expression of the caspase inhibitors p35 or DIAP1 also did not block delamination. The low relevance of apoptosis in Omb-induced cell migration may explain why blocking of the JNK pathway did not promote the delamination phenotype (Fig. 4F and G).

Studies of cancer cell motility using different experimental approaches, including intravital imaging, indicate the existence of two major modes of individual cancer cell migration: Amoeboid and fibroblastic, the former mode being independent of proteolytic activity [78, 80, 120]. Metastatic spread which requires penetration of basement membranes generally depends on extracellular proteolytic acitvity (predominantly matrix metalloproteinases) for local degradation of the ECM [79, 121]. In several experiments in which motility of imaginal disc cells was demonstrated, induction or requirement of MMP1 or MMP2 was observed [42, 75, 89, 110, 122-124]. Also in *Drosophila* transplantation models, systemic spread of cancer cells is blocked by expression of the MMP inhibitor Timp in the target tissue [76]. However, Timp expression did not prevent intraepithelial migration or basal delamination of Omb/TBX2 overexpressing cells (Fig. 8). Also in a standard genetic *Drosophila* cancer model (Act5C>Ras^V12^; *scrib*), Timp co-expression in the transformed cells proved insufficient to prevent metastasis. Co-expression of a second protease inhibitor, RECK, was necessary for repression of metastasis [124]. In invasive growth, MMP expression can be induced in stroma or target cells [125] [76]. It is conceivable that in experimental animals, not only the genotypes of transformed cells and target tissue but also the design of the experiment (co-expression of oncogenes and protease inhibitor vs. spatially separated expression) determines its outcome. In our experiments, Timp proved ineffective in blocking migration even when Omb/TBX2 and Timp were co-expressed in the entire imaginal disc (Fig. 8).

In recent years, the traditional view of metastasis as an endpoint of tumor progression, reached by the accumulation of clonally selected mutations, has been challenged. There are indications that cancer cells may be able to leave the primary tumor early in progression [88, 126-129]. In some cancers, the level or activity of TBX2/3 appear to control cellular motility [3, 7, 13, 19]. This salient role in metastasis makes TBX2/3 an interesting target in cancer therapy [6, 31]. Our data show that an about threefold overexpression of Omb (relative to the peak expression in the medial wing disc) is sufficient to induce invasive cell behavior. Data in the literature on the overexpression of TBX2 and TBX3 in tumor samples and tumor cell lines vary depending on the reference sample chosen and on the molecular category inspected (DNA, RNA, or protein) but tend to lie in the range of 1.5 to 10-fold [2, 14, 130-133]. Importantly, small differences in TBX expression can correlate with drastic differences in patient survival [12]. This suggests that relatively small decreases in TBX2/3 expression or activity may suffice to suppress the pathogenic action of these proteins.

Expression and activity of TBX2/3 are controlled by several signaling pathways [3, 7, 21, 28, 103, 134-139]. The repressor function of TBX2 and TBX3 can be strongly activated by phosphorylation through p38 [103, 134]. Furthermore, numerous protein interaction partners have been identified for TBX2/3 [2, 8, 16, 17, 97, 140-147], some of which are clearly related to the tumorigenic activity of TBX2/3. Hence, abundance or activity of TBX2/3 could potentially be attenuated by small molecules at transcriptional and posttranscriptional levels or by interfering with the interaction with transcriptional co-factors (cf. [148]).

## MATERIALS AND METHODS

### Drosophila stocks

All the stocks are described at http://flybase.bio.indiana.edu unless indicated otherwise. Larvae were reared at 25°C. Larvae containing Gal80^ts^-Gal4 combinations were raised at 18°C and then shifted to 29°C for UAS transgene expression. Transgenes used were UAS-GFP, UAS-bsk[DN] [58], UAS-CD8-GFP [149], 30A-Gal4 [49], AYGal4 [150], C765-Gal4 [56], tubP-Gal80ts [72], UAS-omb [32], UAS-DE-cad and UAS-DE-cad[DN] [69], UAS-DIAP1(Bloomington Stock Center), UAS-Timp [75], puc-lacZ [60]. Viking-GFP is a gene trap line (Collagen IV; G00454, http://flytrap.med.yale.edu). dpp-Gal4 was obtained from K. Basler.

### Construction of UAS-TBX2-Flag transgenic flies

We first constructed a Gateway® (Invitrogen) compatible UAS vector based on the vector pCa4B2G [151]. The UAS cassette was excised from pUAST [49] by BamHI digest and re-inserted into the BamHI site of pCa4B2G to obtain pCa4B2G-UAS. The Gateway cloning cassette, encompassing attR1, chloramphenicol resistance gene, the bacterial cell death gene *ccdB*, and attR2, was obtained from pT-REx-DEST30 (Invitrogen) by BclI linker amplification. This cassette was cloned into the BglII site of pCa4B2G-UAS resulting in pCa4B2G-UAS-GW. The human TBX2 open reading frame was amplified from a cDNA clone (TBX2-pOTB7, Imagenes; NCBI: NCBI: BC052566) by linker-PCR which appended CACC at the 5′ end for directional cloning and and a triple FLAG-tag at the 3′ end (TBX2for: CACCatggcttacca cccgttcCAC, TBX2rev: TCACTACTTGTC ATCGTCATCCTTGTAGTCGATGTCATGAT CTTTATAATCACCGTCATGGTCTTTGTAGTCC TTGGGCGACTCCCGGCC). Because of the high GC content, amplification was perfomed with GC-rich PCR System (Roche). The amplified fragment was polished with PfuUltra^TM^ High-Fidelity DNA polymerase using the PCR Polishing kit (Stratagene) and directionally cloned into pENTR^TM^/D-TOPO® using the pENTR^TM^ Directional TOPO® Cloning Kit (Invitrogen). From this *entry* clone the tagged TBX2 sequence was transferred to the *destination* vector pCa4B2G-UAS-GW by LR recombination using Gateway® LR Clonase^TM^ II emzyme mix (Invitrogen) to obtain TBX2-3xFLAG/pCa4B2G-UAS-GW. Transgenic flies were obtained by phiC31 recombination at attP landing site 58A (Rainbow Transgenic Flies).

### Cell clone generation

Marked clones of mutant cells were generated by Flp-mediated mitotic recombination [152] subjecting first instar larvae to a 35.5°C heat-shock for 30 min. Transgenes were expressed using the Gal4–UAS system [49].

### Immunohistochemistry

Dissected wing imaginal discs were fixed and immunostained using standard procedures for confocal microscopy. Imaginal discs dissected from third instar larvae were fixed and stained with rhodamine phalloidin (1:1000, Cytoskeleton), DAPI (1:500, Sigma), ECM-P1 [53] and appropriate primary antibodies: rabbit anti-Omb (1:1000) [32], rabbit anti-GFP (1:2000) (Clontech), rabbit anti-Cleaved Caspase-3 (Asp175) (1:500) (Cell signalling), mouse anti-GFP (1:1000) (Sigma), mouse anti-Flag (1:200) (Cali-Bio), goat anti-DE-Cadherin (1:200) (Santa Cruz), rabbit anti-Laminin (1:500) (ABCam), mouse anti-N2 7A1 Armadillo (1:10) and mouse anti-Dlg1 (1:10) (DSHB). Secondary antibodies (1:100) (Jackson Immuno Research) used were anti-mouse FITC, anti-mouse Cy5, anti-rabbit FITC, anti-rabbit Cy3, and anti-goat Cy3. Images were recorded on a confocal microscope.

### In situ hybridization

Third instar larvae were dissected in ice-cold 1x PBS and fixed in 4% paraformaldehyde. After several washing steps with PBTween, larvae were incubated in 1ml 1.2 M triethanolamine, pH 7,0 mixed with 2,5 μl acetic anhydride for 1 hour and then prehybridized for 4 hours at 65°C. Larval tissue was hybridized at 65°C overnight with digoxigenin-labeled RNA antisense probes that were generated using a PCR-amplified template tagged with a T7-polymerase (Roche) binding site.

The next day, anti-DIG-antibody labelled with alkaline phosphatase (Roche) was diluted 1 to 1000 and incubated with larval tissue for 2 hours at room temperature. The staining was performed with 3,3 μl NBT and 1,5 μl BCIP solutions in 1ml AP-buffer. To stop the staining, tissue was washed for 10 min in methanol. For further handling, larvae were stored in 70% glycerol in PBS.

## SUPPLEMENTARY MATERIAL FIGURES


