# Supplementary material for: The orthologous Tbx transcription factors Omb and TBX2 induce epithelial cell migration and extrusion in vivo without involvement of matrix metalloproteinases
Source: Oncotarget. 2014 Sep 2;5(23):11998–2015. doi: 10.18632/oncotarget.2426 (PMC4322970; doi:10.18632/oncotarget.2426)
Supplement: Supplementary file 1 [file oncotarget-05-11998-s001.pdf]

The orthologous Tbx transcription factors Omb and TBX2 induce epithelial cell migration and extrusion *in vivo* without involvement of matrix metalloproteinases

Supplementary Material

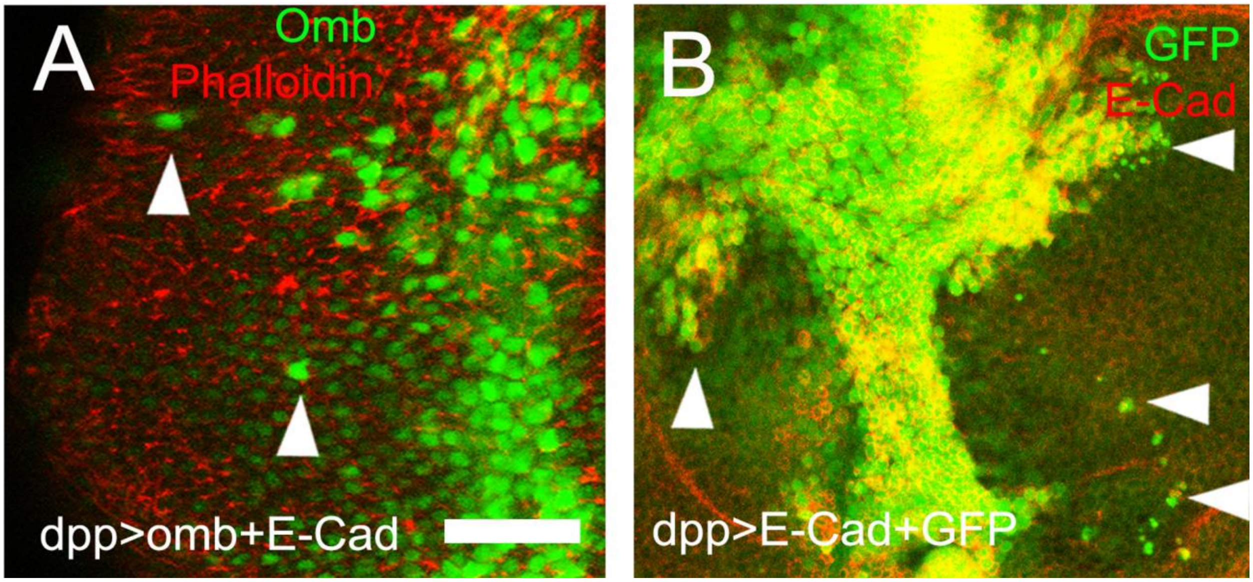

Figure S1: DE-cad overexpression is sufficient to induce cell migration. (A) Cells co-overexpressing Omb (green) and wild-typic DE-cad in the dpp-Gal4 domain could migrate long distance (arrowheads). The disc is counterstained with rhodamine phalloidin (red). (B) Cells overexpressing wild-typic DE-cad (red) in the dpp-Gal4 domain (marked by co-expression of GFP, green) could migrate to the disc periphery (arrowheads).

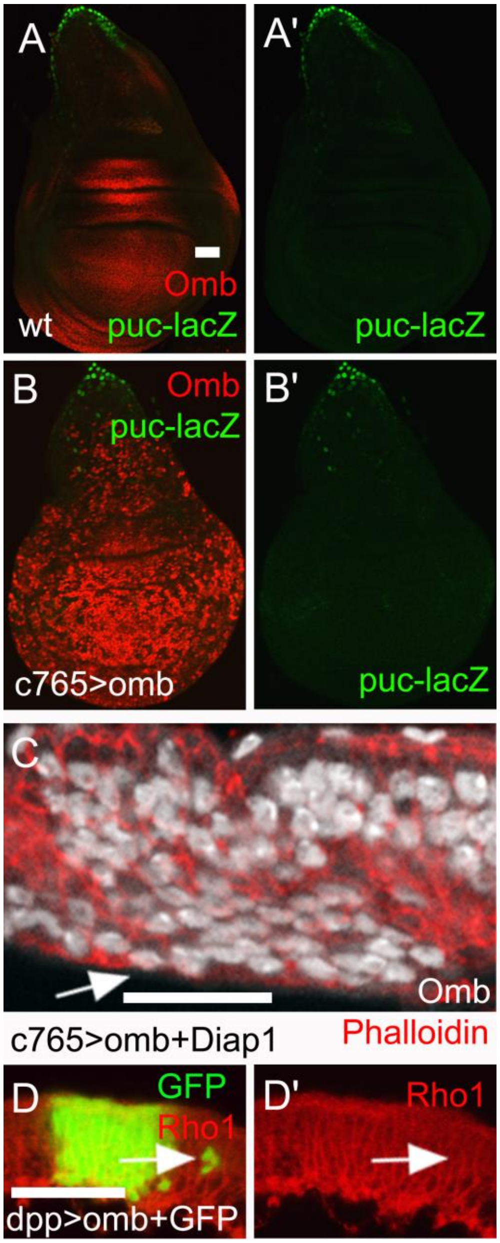

Figure S2: Activation of JNK signaling and apoptosis are not required for Omb-induced cell motility. Puc-lacZ is induced by JNK signaling. In wildtype (A) and in wings uniformly overexpression Omb (red) (B) puc-lacZ (green) was restricted to the most proximal part of the disc (top) where it functions in the later fusion of the two wing discs along the dorsal midline. (C) Co-expression of Omb (white) along with the general caspase inhibitor DIAP1 did not prevent cell motility (arrow). The disc is counterstained with rhodamine phalloidin (red). (D) *Omb* overexpression (marked by GFP, green) did not affect the level of Rho1 (red).

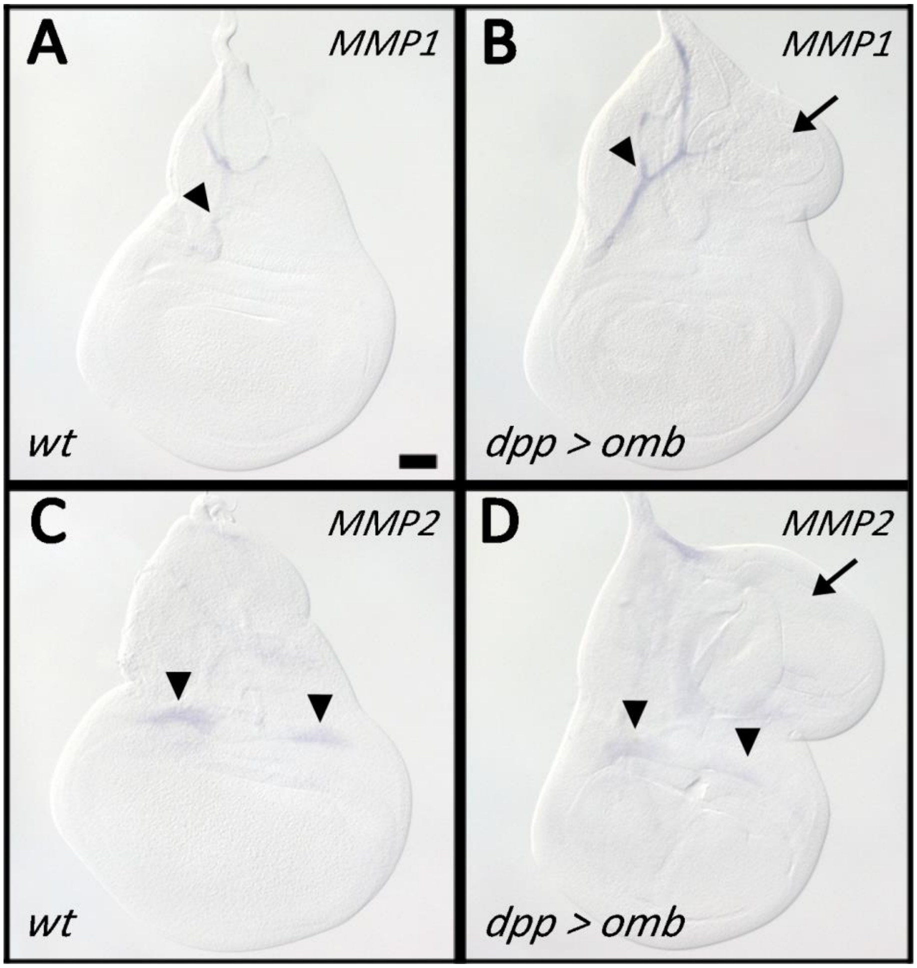

Figure S3: ***MMP1*** **and** ***MMP2*** **are not induced by** ***omb*** **overexpression.** ***MMP*** **expression was visualized by** ***in situ*** **hybridization.** (A) and (C) *wild type*. (B) and (D) *omb* overexpression by dpp>omb causes the outgrowth of a second wing from the notum (arrow) [32]. dpp>omb and control flies were reared at 25°C and then transferred to 29.8°C 36h before dissection. (A, B) *MMP1* was mainly expressed in a tracheal branch adhering to the wing disc (arrowhead). (C, D) *MMP2* was expressed in a disrupted stripe along the dorsal hinge (arrowheads). The expression of neither gene was enhanced by dpp>omb.
